# G-protein coupled estrogen receptor activation protects the viability of hyperoxia-treated primary murine retinal microglia by reducing ER stress

**DOI:** 10.18632/aging.103733

**Published:** 2020-09-13

**Authors:** Rong Li, Yao Wang, Pei Chen, Jiamin Meng, Hongbing Zhang

**Affiliations:** 1 Department of Ophthalmology, The First Affiliated Hospital of Xi'an Medical University, Xi’an 710077, Shaanxi, PR China; 2 Department of Ophthalmology, Eye Institute of Shaanxi Province and Xi'an First Hospital, Xi'an 710002, Shaanxi, PR China; 3 School of Life Sciences, Northwest University, Xi’an 710069, Shaanxi, PR China

**Keywords:** G-protein coupled estrogen receptor, retinopathy of prematurity, hyperoxia, endoplasmic reticulum stress, microglia

## Abstract

In this study, we investigated the effects of G-protein coupled estrogen receptor (GPER) activation in the early phase of retinopathy of prematurity (ROP) and its association with endoplasmic reticulum (ER) stress using primary murine retinal microglia as an experimental model. Fluorescence microscopy results show that the CD11c-positive primary retinal microglia *in vitro* cultured for 14 days were GPER-positive. GPER activation using GPER-agonist G-1 reduced autophagy and increased the viability of the hyperoxia-treated primary murine retinal microglia. Furthermore, GPER activation reduced the expression of ER stress-related proteins, IRE1α, PERK and ATF6 in the hyperoxia-treated primary murine retinal microglia compared to the corresponding controls. GPER activation significantly reduced a time-dependent increase in IP3R-dependent calcium release from the ER, thereby maintaining higher calcium levels in the ER of hyperoxia-treated primary retinal microglia. However, the protective effects of G-1 on the hyperoxia-treated primary retinal microglia were eliminated by inactivation of GPER using the GPER-antagonist, G-15. In conclusion, our study demonstrates that GPER activation enhances the survival of hyperoxia-treated primary retinal microglia by reducing ER stress. Our study demonstrates the therapeutic potential of GPER agonists such as G-1 in the early phase of ROP.

## INTRODUCTION

Retinopathy of prematurity (ROP) is the leading cause of childhood blindness because of abnormal vascular development in the retina of preterm infants [1]. The hypoxic environment in the uterus is essential for the natural development of vasculature, nervous system, and other organs [2, 3]. Premature birth exposes the preterm newborns to an oxygen-rich environment and tissues may experience oxidative stress because of excessive production of reactive oxygen species (ROS) or reactive nitrogen oxide species (RNOS). The murine and rat models of oxygen-induced retinopathy (OIR) demonstrate that oxidative stress causes ROP via dysregulation of several signaling pathways [4–6]. Therefore, alleviation of oxidative stress is critical for preventing ROP in preterm infants [7]. Premature birth also terminates the supply of the maternal hormone, estrogen [8], which drives proliferation and differentiation of mammalian organs [9]. Several studies demonstrate that exogenous administration of estrogen or estrogen agonists promote retinal vascular development, thereby suggesting potential therapeutic avenue to prevent or alleviate ROP [10–13]. In our previous study, we showed that treatment with the estrogen receptor agonist 17β-estradiol significantly reduces oxidative stress in the hyperoxic phase of OIR [11].

G-protein coupled estrogen receptor (GPER) or GPR30 is a transmembrane estrogen receptor that binds with high affinity to 17β-estradiol [14]. GPER is localized to the endoplasmic reticulum (ER), where it specifically binds estrogen and regulates ER functions during normal physiological conditions and estrogen-related human diseases [15, 16]. Oxidative stress induces protein unfolding because of oxidative protein damage and triggers the unfolding protein response (UPR) in the ER [17]. GPER in combination with estrogen rapidly reduces ER stress by decreasing ROS levels [15, 16]. The expression of GPER in the retina has been reported [14], but, its activation by estrogen or estrogen agonists to alleviate ROP has not been reported. Moreover, the functional relevance of GPER in ER stress mechanisms during hyperoxia-induced ROP is not clear.

Microglia is one of the primary immune cell types that reside in the retina and are constantly engaged in the immune surveillance [18]. Activation of microglia involves a complex interplay between different retinal cell types and diverse signaling pathways. Microglia populate the retina at an early embryonic age, well before the astrocytes and the development of the retinal vasculature [19]. Recent evidence suggests that microglia play a significant role in retinal development, function, and diseases [18, 20]. The retinal microglia influence vascular branching density and endothelial cell proliferation [21, 22]. They are also implicated in the pathology of diabetic retinopathy and OIR [23, 24]. In this study, we investigated the protective effects of GPER activation on hyperoxia-treated primary murine retinal microglia. We also explored the effects of GPER activation on hyperoxia-induced ER stress in the retinal microglia in order to elucidate potential therapeutic mechanisms of estrogen agonists during the early phase of ROP.

## RESULTS

### Morphology and GPER expression in murine primary retinal microglia

**Figure 1:**
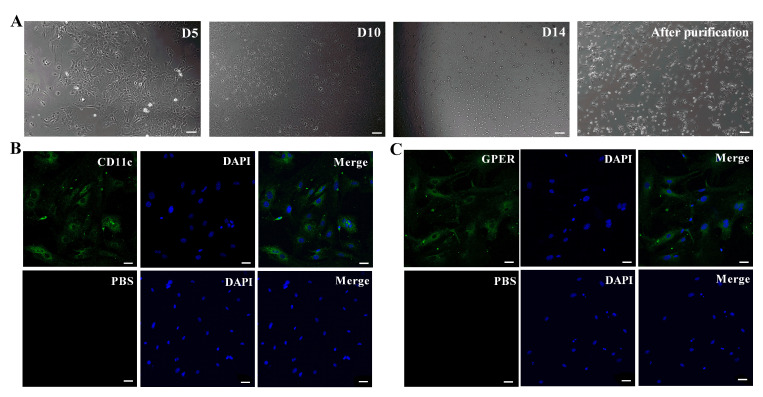
**Basic characterization of primary murine retinal microglia.** (**A**) The representative images (100×, Bar=10μm) show *in vitro* cultured primary retinal microglia cells on days 5 (D5), 10 (D10) and 14 (D14). (**B**) Representative confocal fluorescence microscopic images (400×; Bar=25μm) show Alexa Fluor 488-tagged anti-CD11c antibody (green) staining of primary murine retinal microglia. The negative control cells are treated with PBS instead of the primary anti-CD11c antibody. (**C**) Representative confocal fluorescence microscopic images (400×; Bar=25μm) show Alexa Fluor 488-tagged anti-GPER antibody (green) staining of primary murine retinal microglia. The negative control cells are treated with PBS instead of the primary anti-GPER antibody.

The purified primary murine retinal microglia showed highly refractive short rod-shaped morphology with few branches or processes protruding from their ends, when observed under a light/phase-contrast microscope after 14 days of *in vitro* culture (Figure 1A). Fluorescence microscopy results showed that the primary murine retinal microglia were CD11c-positive (Figure 1B) and GPER-positive (Figure 1C). The average fluorescent intensity was 23.37±3.79 for CD11c and 11.72±1.69 for GPER (Figure 1B, 1C).

### Activation of GPER increases the viability of primary retinal microglia under hyperoxia

**Figure 2:**
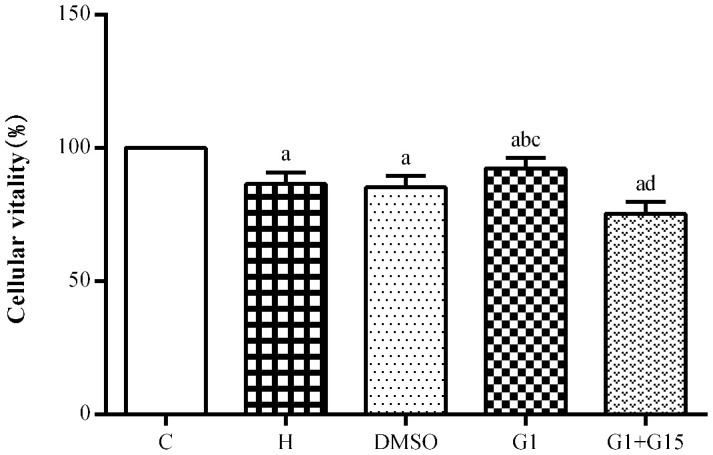
**GPER activation increases the viability of hyperoxia-treated primary retinal microglia.** CCK-8 assay results show the viability of primary retinal microglial cells in the (A) control (C), (B) hyperoxia (H), (C) hyperoxia+DMSO (DMSO), (D) hyperoxia+G-1 (G-1), and (E) hyperoxia+G-1+G-15 (G-1+G-15) groups. Note: *P*<0.05 indicates statistical significance. The experiment was repeated thrice.

CCK-8 assay showed that the viability of hyperoxia-treated primary retinal microglia in the H, DMSO, G-1 and G1+G-15 groups was significantly reduced at 48 h compared to the control group (all *P*<0.05; Figure 2). Furthermore, under hyperoxia conditions, the viability of the G-1 group was significantly higher than those in the H and DMSO groups (both *P*<0.05; Figure 2). Moreover, viability of the G-1+G-15 group was significantly lower compared to the G-1 group (*P*<0.05; Figure 2). These results demonstrate that GPER activation increases viability of the hyperoxia-treated primary retinal microglia.

### GPER activation reduces cellular apoptosis in hyperoxia-treated primary retinal microglia

**Figure 3:**
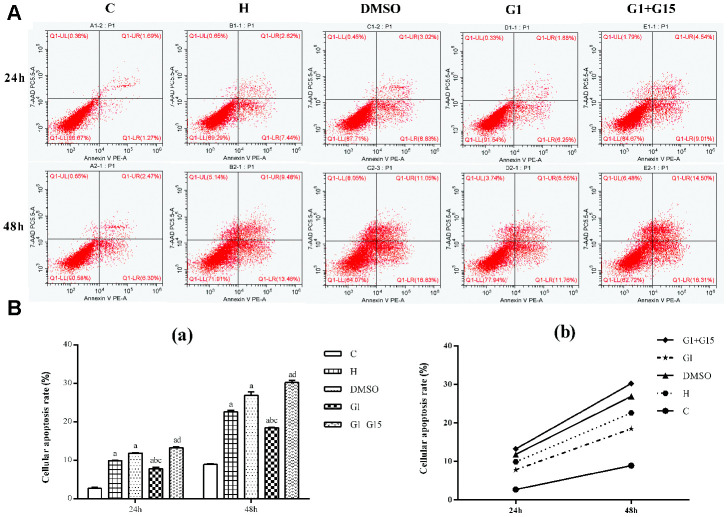
**GPER activation reduces apoptosis of hyperoxia-treated primary retinal microglia.** (**A**) Representative FACS plots and (**B**) Histograms show the percent apoptotic cells of primary retinal microglial cells in the (a) control (C), (b) hyperoxia (H), (c) hyperoxia+DMSO (DMSO), (d) hyperoxia+G-1 (G-1), and (e) hyperoxia+G-1+G-15 (G-1+G-15) groups at 24 and 48 h. The cells were stained with Annexin V-PE and 7-AAD. The percentage of apoptotic cells included Annexin-V^+^ AAD^+^ plus Annexin-V^+^ AAD^-^ cell. *P*<0.05 denotes statistical significance. The experiment was repeated thrice.

Flow cytometry analysis shows that the apoptotic rate was significantly higher in the hyperoxia-treated primary retinal microglia in the H, DMSO, G-1 and G1+G-15 groups compared to the control group at both 24 h and 48 h (all *P*<0.05; Figure 3). Furthermore, the apoptotic rate in the G-1 group was significantly lower at both 24 and 48 h compared to the H and DMSO groups (all *P*<0.05; Figure 3). Moreover, the apoptotic rate was significantly higher in the G-1+G-15 group at 24 and 48 h compared to the G-1 group (all *P*<0.05; Figure 3A). We also observed that apoptotic rate was higher in all groups at 48 h than at 24 h (Figure 3B). These results demonstrate that GPER activation reduces apoptosis in hyperoxia-treated microglia.

### GPER activation reduces autophagy in hyperoxia-treated primary retinal microglia

**Figure 4:**
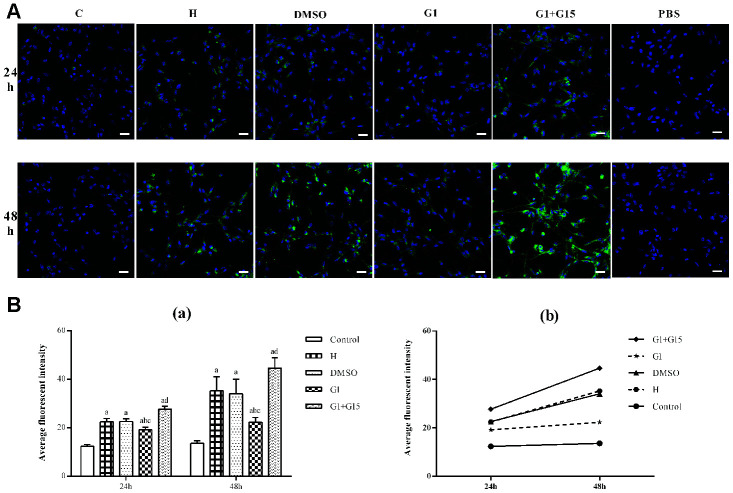
**GPER activation reduces autophagy in hyperoxia-treated primary retinal microglia.** (**A**) Representative confocal fluorescence images (400×, Bar=25μm) show Cyto-ID labeled autophagic vesicles (green) in the (a) control (C), (b) hyperoxia (H), (c) hyperoxia+DMSO (DMSO), (d) hyperoxia+G-1 (G-1), and (e) hyperoxia+G-1+G-15 (G-1+G-15) groups of primary retinal microglia at 24 and 48 h. The negative control cells were treated with PBS instead of Cyto-ID. (**B**) Histogram plots show average fluorescence staining intensity (Cyto-ID; green) at 24 and 48 h in the (a) control (C), (b) hyperoxia (H), (c) hyperoxia+DMSO (DMSO), (d) hyperoxia+G-1 (G-1), and (e) hyperoxia+G-1+G-15 (G-1+G-15) groups at 24 and 48 h. *P*<0.05 denotes statistical significance. The experiments were repeated thrice and means±S.D are shown.

Next, we analyzed the status of autophagy in the hyperoxia-treated primary retinal microglia. We observed increased autophagy in hyperoxia-treated primary retinal microglia from the H, DMSO, G-1 and G1+G-15 groups compared to the control group at 24 and 48 h (all *P*<0.05; Figure 4A, 4B). Furthermore, the G-1 group showed significantly lower autophagy at 24 and 48 h compared to the H and DMSO groups (all *P*<0.05; Figure 4A, 4B). Moreover, autophagy was significantly increased in the G-1+G-15 group at 24 and 48 h compared to the G-1group (all *P*<0.05; Figure 4A, 4B). We also observed that autophagy in hyperoxia-treated primary retinal microglia from all groups was higher at 48 h compared to 24 h (Figure 4B). These results demonstrate that GPER activation reduces autophagy in hyperoxia-treated microglia.

### GPER activation reduces ER stress in hyperoxia-treated primary retinal microglia

**Figure 5:**
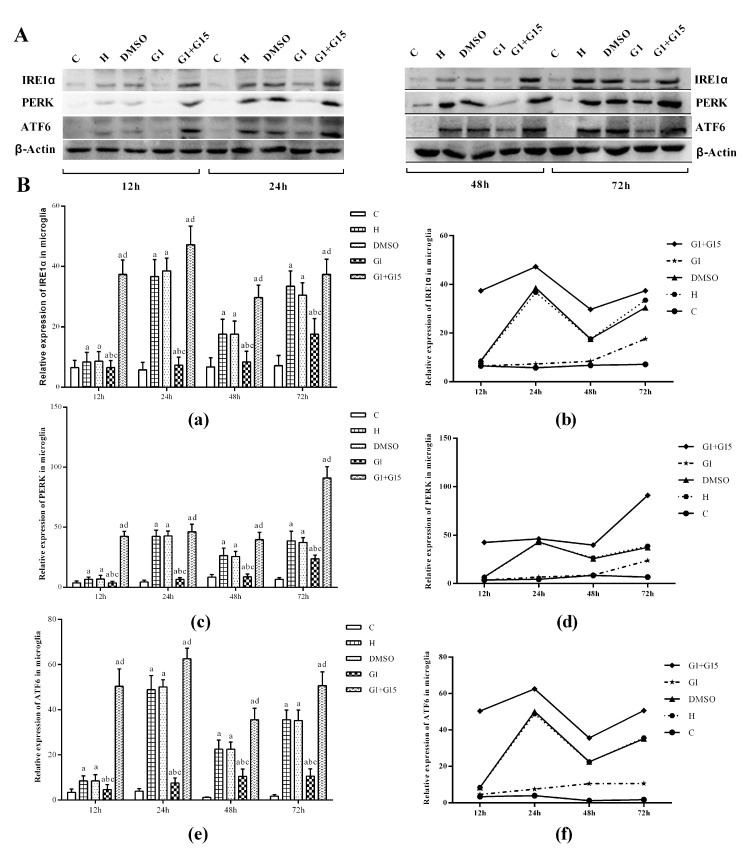
**GPER activation reduces induction of ER-stress related proteins in hyperoxia-treated primary retinal microglia.** (**A**) Representative western blot images and (**B**) Histogram plots show the levels of IRE1α, PERK and ATF6 proteins in the (a) control (C), (b) hyperoxia (H), (c) hyperoxia+DMSO (DMSO), (d) hyperoxia+G-1 (G-1), and (e) hyperoxia+G-1+G-15 (G-1+G-15) groups of primary retinal microglia at 12h, 24h, 48h and 72h. β-actin was used as internal control. *P*<0.05 denotes statistical significance. The experiments were repeated thrice and means±S.D are shown.

Western blot analysis showed that the levels of ER stress proteins, IRE1α, PERK and ATF6 were significantly higher in the primary retinal microglia belonging to H, DMSO, G-1 and G1+G-15 groups compared to the control group at 12, 24, 48 and 72 h (all *P*<0.05; Figure 5). Furthermore, the levels of IRE1α, PERK and ATF6 proteins were significantly lower in the G-1 group compared to the H and DMSO groups (all *P*<0.05; Figure 5). Moreover, the levels of IRE1α, PERK and ATF6 proteins were significantly lower in the G-1 group compared to the G-1+G-15 group (all *P*<0.05; Figure 5A, 5B). We also observed that the levels of IRE1α, PERK and ATF6 changed dynamically over time in all hyperoxia-treatment groups. In the H, DMSO, and G-1+G-15 groups, IRE1α, PERK and ATF6 levels increased at 24 h, then reduced at 48 h, and again increased at 72 h (Figure 5B). In the G-1 group, the expression of IRE1α and PERK incrementally increased from 24-72 h, whereas, the expression of ATF6 increased gradually at 24 and 48 h, but slightly decreased at 72 h (all *P*<0.05; Figure 5B). These results demonstrate that GPER activation reduces ER stress in hyperoxia-treated primary retinal microglia.

### GPER activation decreases IP3R activity and increases ER calcium levels in hyperoxia-treated primary retinal microglia

**Figure 6:**
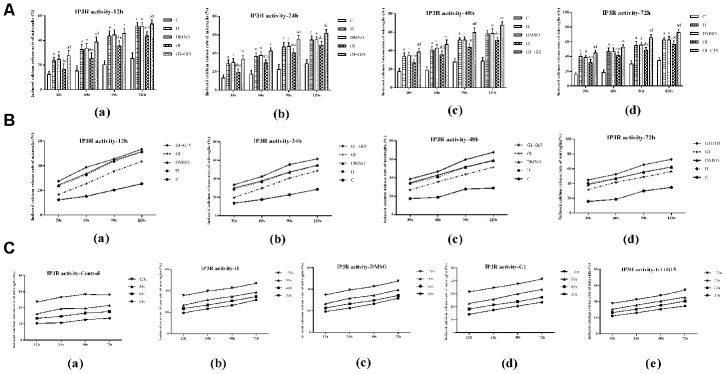
**GPER activation reduces hyperoxia-induced IPER-dependent calcium release from the ER in primary retinal microglia.** (**A**) The bar graphs show the rates of calcium release from the ER at 12, 24, 48 and 72 h in the (a) control (C), (b) hyperoxia (H), (c) hyperoxia+DMSO (DMSO), (d) hyperoxia+G-1 (G-1), and (e) hyperoxia+G-1+G-15 (G-1+G-15) groups of primary retinal microglia. (**B**) The curves show the IPER-dependent calcium release rates between 30 s to 120 s from the ER for each of the five treatment groups of primary retinal microglia at 12, 24, 48 and 72 h. (**C**) The curves show the calcium release rates from the ER in all 5 groups of primary retinal microglia between 12-72 h. *P*<0.05 denotes statistical significance. The experiments were repeated thrice and means±S.D are shown.

**Figure 7:**
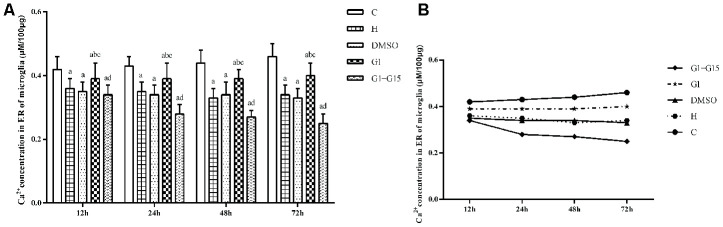
**GPER activation maintains ER calcium concentration in the hyperoxia-treated primary retinal microglia.** (**A**) The bar graphs show the ER calcium concentrations at 12, 24, 48 and 72 h in the (a) control (C), (b) hyperoxia (H), (c) hyperoxia+DMSO (DMSO), (d) hyperoxia+G-1 (G-1), and (e) hyperoxia+G-1+G-15 (G-1+G-15) groups of primary retinal microglia. (**B**) The curves show the change in ER calcium concentration in each of the five groups of primary retinal microglia between 12-72h. *P*<0.05 denotes statistical significance. The experiments were repeated thrice and means±S.D are shown.

Next, we analyzed the effects of GPER activation on the IP3R-dependent calcium release in the ER of hyperoxia-treated primary retinal microglia. IP3R-dependent calcium release was significantly higher in the hyperoxia-treated H, DMSO, G-1 and G-1+G-15 groups compared to the control group at 12, 24, 48, and 72 h (all *P*<0.05; Figure 6). Furthermore, IP3R-dependent calcium release was significantly lower in the G-1 group compared to the H and DMSO groups (all *P*<0.05; Figure 6). Moreover, IP3R-dependent calcium release was significantly higher in the G-1+G-15 group compared to the G-1 group (all *P*<0.05; Figure 6A). We also observed that within each of the five groups, the induced calcium release rate gradually increased between 30 s and 120 s (Figure 6B) and between 12 to 72 h (Figure 6C). Therefore, the calcium concentration in the microglial ER of all hyperoxia-treatment groups decreased gradually at 12, 24, 48, and 72 h. The total calcium concentration in the ER was significantly lower in the H, DMSO, G-1 and G-1+G-15 groups compared to the control group (all *P*<0.05; Figure 7). The calcium concentration in the ER of the G-1 group was significantly higher than in the H and DMSO groups at all time points (all *P*<0.05; Figure 7). The calcium concentration in the ER of G-1+G-15 group microglia was lower than the ER of G-1 group microglia at all time points (all *P*<0.05; Figure 7A). We also observed that the ER calcium concentration within the five groups was relatively stable at all time points except for the G-1+G-15 group, which showed a slight decrease at 24h and then remained relatively stable (Figure 7B). These results demonstrate that GPER activation reduces the IP3R activity in the hyperoxia-treated primary retinal microglia to maintain a relatively stable calcium concentration in the ER.

## DISCUSSION

The survival rates of infants with extremely low birth weight has improved significantly in the recent decades because of improvements and advances in the neonatal intensive care [25]. However, concurrently, the incidence rates of ROP have also increased. The two phases in the pathophysiology of ROP include hyperoxia-related vaso-obliteration of the immature retina in phase 1 and abnormal vasoproliferation due to retinal hypoxia in phase 2 [26]. The current treatment modalities such as laser therapy, intravitreal injection of anti-VEGF antibodies, scleral buckling or vitrectomy are associated with a high incidence of side effects, treatment failure or disease recurrence [27, 28]. Moreover, the current treatment strategy focuses more on the retinal neovascularization in phase 2 [29] and does not address substantially the primary cause of ROP, which involves hyperoxia-related arrest of retinal development in phase 1. Therefore, understanding the effects of hyperoxia on the normal retinal development is essential for devising effective treatment modalities for ROP. In this study, we aimed to investigate the effects of hyperoxia on the survival of primary murine retinal microglia as well as its effects on ER stress. We also addressed the protective effects of GPER activation on hyperoxia-related ER stress and related mechanisms in the primary retinal microglia. We exposed primary retinal microglia to 75% O_2_ to simulate conditions of hyperoxia in phase 1 of ROP.

Estrogen is an important endocrine hormone that plays an important role in human development and disease [30]. The vasodilatory function of estrogen improves the retinal blood flow and can protect against several retinal pathologies [31]. Several studies demonstrate that estrogen is a potential treatment for ROP, but, the mechanisms through which estrogen protects against ROP is not well established [10–12]. GPER is an ER-resident receptor for 17β-estradiol (predominant and most potent endogenous estrogen), and is expressed in several human tissues [32]. GPER is considered as a novel therapeutic target and prognostic indicator for many human diseases related to the reproductive, nervous, endocrine, immune, and cardiovascular systems [15]. In view of the important role of microglia in retinal development, function, and diseases [18, 20], we investigated the effects of GPER activation on their survival under hyperoxic conditions. Our study demonstrates that exposure to hyperoxia significantly reduces the viability of the primary retinal microglia compared to normoxia-treated controls. This suggests that retinal microglia undergo apoptosis during the early stages of ROP. Furthermore, hyperoxia treatment induces autophagy, a stress-related alternate pathway of cell-death [33], was consistent with the trend of apoptosis of microglia under hyperoxia. Impaired autophagy has been reported in the oxygen-induced retinopathy (OIR) model mice [34]. However, it is not known whether microglial autophagy contributes to OIR or ROP. In several human diseases, autophagy may act as a ‘double-edged sword’; in some cases, autophagy may help cells to survive by acting as a mechanism that is necessary to overcome stress conditions, whereas, in other instances, autophagy may serve as an alternate mechanism of cell death [35, 36]. Our study demonstrates that hyperactivation of autophagy in the hyperoxia-treated primary retinal microglia promotes cellular apoptosis. We also demonstrate that the GPER agonist, G-1, increases viability of the microglia, whereas, pre-treatment with GPER antagonist, G15, reduces the beneficial effects of G1 on hyperoxia-treated primary retinal microglia. Furthermore, as previously reported for cardiocytes and neurons [37, 38], our study demonstrates that GPER activation protects retinal microglia against hyperoxia-induced cell death. This suggests that GPER activation may promote retinal vascular development during the early stages of ROP.

The neuroprotective action of estrogen is partly associated with its antioxidant activity [39, 40]. Endoplasmic reticulum (ER) is an important organelle that mediates new protein synthesis, new and unfolded protein folding, and post-translational modifications that are necessary for the production of functional cellular proteins. The unfolded protein response (UPR) is activated in the ER upon oxidative stress and is necessary to restore cellular homeostasis by increasing the ability of ER to fold the unfolded or misfolded proteins [17]. Activation of the three ER transmembrane proteins, IRE1α, PERK and ATF6, is essential in initiating UPR upon ER stress. Persistent ER stress and uncontrolled UPR initiates apoptosis and contributes to the pathogenesis of several human diseases [41, 42]. In the present study, we demonstrate that hyperoxia induces ER stress in the retinal microglia as shown by elevated levels of IRE1α, PERK and ATF6 proteins compared to the normoxic control. Moreover, the activation of GPER by G-1 reduces the ER stress in the retinal microglia and is consistent with findings reported in cerebral ischemia/reperfusion injury and glucotoxicity induced-pancreatic beta-cell death [38, 43]. IP3R is located on the ER surface and is responsible for the regulation of calcium release from the ER [44]. There is close interaction for calcium exchange between the ER and the mitochondria [45]. Accumulation of calcium in the mitochondria causes excessive production of reactive oxygen species (ROS), which upregulates UPR in the ER and contributes to calcium leakage from the ER [46]. In this study, we demonstrate that hyperoxia increases the calcium transfer activity of the IP3R, thereby reducing the calcium concentration in the ER, which is consistent with previous studies regarding the role of the IP3R-Ca^2+^ axis during the oxidative stress [47]. We also demonstrate that the activation of GPER reduces the IP3R activity and the subsequent leaking of calcium to the mitochondria. It helps maintain a stable concentration of calcium in the ER. These results suggest that activation of GPER helps maintain calcium homeostasis in the ER under the stress of hyperoxia.

In conclusion, our study demonstrates that activation of GPER protects the survival of retinal microglia during hyperoxia, which mimics early stages of ROP. Our results suggest that reducing ER stress through GPER activation is a potential therapeutic strategy for ROP. However, the effects of GPER activation on retinal vascular development need to be further verified in studies using OIR models and in ROP patients.

## MATERIALS AND METHODS

### Isolation, purification and culturing of primary murine retinal microglia

The animal-based experiments were all approved by the Institutional Animal Care and Use Committee and the Ethical Committee of Xi’an Medical University, and conducted according to the Statement for the Use of Animals in Ophthalmic and Vision Research. We sacrificed 3-day-old C57BL/6J mice obtained from the Experimental Animal Center of Xi’an Medical University by cervical dislocation. We isolated the eyes and then carefully cut open from the limbus circularly. The fragile retinas, which are about 1 mm × 1 mm in size, were separated out from the corneas, sclera and lens in a sterile state. The retinas were washed with DMEM-F12 medium (Gibco, Carlsbad, CA, USA) without serum thrice, and then digested with trypsin (Beyotime, Shanghai, China) for 15 min with constant shaking. Then, complete culture medium (DMEM-F12 medium with 10% serum) was added to the retinal cell suspension and centrifuged at 1000 rpm/min for 5min in a new sterile 15mL centrifuge tube (Axygen, Union City, CA, USA). After discarding the supernatant, the retinal cells were resuspended in DMEM-F12 medium with 10% serum and cultured in a 25cm^2^ culture bottle (Costar, Cambridge, MA, USA) at 37°C and 5% CO_2_ in a humidified incubator. The culture medium was replaced for the first time after 16 h and again at 3 day intervals. The cell growth was monitored using a inverted phase contrast microscope (Nikon, Tokyo, Japan).

After 14 days of culture, we observed the cell stratification under an inverted phase contrast microscope. At the bottom of the flask, we observed astrocytes as flat, star shaped cells with multiple long projections, whereas, the microglia are attached to the surface of astrocytes and are smaller, circular cells with strong refractivity. We removed the medium and then incubated the cells with 3mL of 0.05% trypsin with gentle shaking to detach the microglia from the astrocytes. Then, we transferred the detached microglia into a 10mL centrifuge tube and added complete medium immediately. Then, we centrifuged the microglial cell suspension. The microglial cell pellet was then resuspended in complete medium and cultured in 24-well plates (Costar, Cambridge, MA, USA) with cover glass at 37°C and 5% CO_2_ for 24 h. Then, after confirming the purity of the microglia, we further cultured them as previously described [48, 49].

### Immunofluorescence

We verified the purity of primary murine retinal microglia cultures and the localization of GPER in the microglia using immunofluorescence. Briefly, the retinal microglial cells were fixed with 4% paraformaldehyde, and then permeabilized with 0.5% Triton X-100 at room temperature for 15 min. Then, the cells were incubated with 6% normal goat serum (Hyclone, Logan, Utah, USA) at room temperature for 30 min for blocking. The slides were then incubated overnight with either mouse anti-CD11c antibody (1:200, Abcam, Cambridge, UK), a microglia specific marker located on their cell membrane [50], rabbit anti-GPER antibody (1:200, Abcam, Cambridge, UK), or PBS (negative control) at 4°C. The cells were then incubated with the Alexa Fluor 488-tagged secondary antibody (1:800, Abcam, Cambridge, UK) for 30 min at 37°C in the dark. Then, after staining the nuclei with DAPI (Beyotime, Shanghai, China), the slides were washed thrice with PBS to remove the excess DAPI. The slides were sealed with anti-fluorescence quencher (Beyotime, Shanghai, China) and imaged using a laser confocal fluorescence microscope (Leica, Solms, Germany). The average fluorescent intensity was measured using the Image J software (version 1.46, National Institutes of Health, Bethesda, MA, USA).

### Cell treatments and grouping

We incubated 4×10^5^ primary retinal microglia in 6-well plates (Costar, Cambridge, MA, USA) at 37°C and 5%CO_2_. When they were fully attached, we divided them into 5 treatment groups, namely, control (C), hyperoxia (H), hyperoxia+DMSO (DMSO), hyperoxia+G-1 (G-1), and hyperoxia+G-1+G-15 (G-1+G-15). The control group was cultured under normoxia conditions. The DMSO group was grown in complete medium with 0.01% DMSO (Amresco, Solon, OH, USA). The G-1 group was treated with 0.1μM G-1 [51], a GPER agonist (MCE, NJ, USA) for half an hour. The G-1+G-15 group was first treated the cells with 1μM G-15 [51], a GPER antagonist (Cayman, Ann Arbor, Michigan, USA) followed by 0.1μM G-1 for another half an hour. Finally, the plates in groups H, DMSO, G-1 and G-1+G-15 were incubated at 37°C and 75% O_2_ to induce hyperoxia for 12 h, 24 h, 48 h or 72 h. The 75% O_2_ concentration for hyperoxia was similar to that chosen for the *in vivo* mouse OIR model [52].

### CCK-8 assay

The viability of retinal microglia was analyzed using the CCK-8 kit (Sigma, St. Louis, MO, USA) as described previously [53]. The blank, control and experimental groups of primary retinal microglia were cultured in DMEM-F12 medium containing 10% FBS, 10mg/L bFGF and 1% penicillin-streptomycin (Gibco, Carlsbad, CA, USA) for 48 h. Then, 10 μl of CCK-8 reagent was added to all wells and the cells were further incubated for another 2 h. Then, the absorbance (A) of each well was estimated at 450 nm in a plate reader. The cell viability of each experimental group (%) was calculated as [A_experiment group_ – A_blank group_] / [A_control group_ -A_blank group_] × 100, where, A_experiment group_ refers to the absorbance value of treated cells, A_blank group_ refers to the absorbance value of medium without cells, and A_control group_ refers to the absorbance value of untreated cells.

### Apoptosis assay

We measure the apoptotic rate of hyperoxia-induced primary retinal microglia by flow cytometry. Briefly, the control and experimental groups of microglia were cultured in 6-well plates for 24 or 48 h. Then, the cells were stained using the Annexin V-PE/ 7-AAD cell apoptosis detection kit (BD, Franklin Lakes, NJ, USA) according to a protocol described previously [54]. The percentage apoptosis in each group was measured by flow cytometry using a CytoFlex flow cytometer (Beckman, USA).

### Autophagy assay

Microglial autophagy was estimated using the Cyto-ID® autophagy detection kit (Enzo, Farmingdale, NY, USA) as previously described [55]. Briefly, the control and experimental groups of primary retinal microglia were washed twice with the assay buffer and incubated with the Cyto-ID stain or PBS (unstained control) and Hoechst 33342 (DNA staining dye) at 37°C for 30 min. The cells were then fixed with 4% paraformaldehyde at room temperature for 15 min, washed again with the assay buffer and immediately imaged using a laser confocal fluorescence microscope. The average fluorescent intensity (integrated density/ area) in each group was measured using the Image J software version 1.46 (National Institutes of Health, Bethesda, MA, USA).

### Western blotting

The microglia from the control and experimental groups (1×10^7^ cells/mL) were homogenized in the RIPA buffer (Beyotime, Shanghai, China). The protein concentrations were quantified using the BCA assay (Pierce, Rockford, IL) according to the manufacturer’s instructions. Then, 40 μg of whole cell protein lysates were boiled in 5μl sample buffer for 5 min followed by separation of the proteins by sodium dodecyl sulfate-polyacrylamide gel electrophoresis (SDS-PAGE). The separated proteins were transferred onto nitrocellulose membranes. The membranes were blocked with 5% non-fat dry milk in TRIS-buffered saline (TBS) for 1h and incubated overnight at 4^o^C with primary antibodies, namely, rabbit anti-IRE1α (1:500, Abcam, Camb, UK), rabbit anti-PERK (1:500, Abcam, Camb, UK), and rabbit anti-ATF6 (1:500, Abcam, Camb, UK), and mouse β-actin (1:1000, Abcam, Camb, UK). Then, after washing thoroughly, we incubated the blots with HRP-conjugated secondary antibodies (1:1000, Sigma, St. Louis, MO, USA) at room temperature for 2 h. The blots were developed using the Enhanced Chemiluminescence (ECL) kit (Cell Signaling Technology, Boston, USA). The levels of IRE1α, PERK and ATF6 proteins relative to β-actin expression were quantified using the Image J software (NIH, Bethesda, USA).

### IP3R activity and calcium concentration in the ER of primary retinal microglia

The activity of inositol-1,4,5-triphosphate receptor (IP3R), which is one of the calcium (Ca^2+^) release channels in the ER membrane [44] was determined using the IP3R functional fluorescence detection kit (Shanghai Yu Duo Biotech Ltd, China). This kit contains a low calcium affinity fluorescent probe, Mag-Fluo-AM, which specifically accumulates in the ER based on the changes in the calcium concentration in the ER. Therefore, Mag-Fluo-AM is used to evaluate the activity of IP3R [34]. The relative fluorescence unit (RFU) was obtained dynamically at 30s, 60s, 90s, and 120s using a fluorescence microplate reader (Bio-Tek ELX800, Winooski, USA). IP3R activity was calculated as the induced calcium release rate at different time points within two minutes. The induced calcium release rate (%) was calculated as [RFU (ER calcium)-RFU (induced calcium release)] / [RFU (ER calcium)-RFU (complete calcium release)] ×100. The total calcium concentration in the ER was estimated using the GENMED ER calcium concentration assay kit (Shanghai Yu Duo Biotech Ltd, China), which measures the fluorescence intensity of Mag-Fluo-AM relative to calcium levels in the ER. The total calcium concentration in the ER (μM/100 μg) was calculated as [RFU (sample) – RFU (blank control)] / RFU (maximum control well) – RFU (sample)] ×22.

### Statistical analysis

The statistical data was analyzed using the SPSS 25.0 software (IBM, USA). All values in this study are presented as means ± standard deviation (SD) of at least three independent experiments. Statistical differences between different experimental and control groups was estimated by multiple-factor repetitive measurement and analysis of variance, pairwise *T*-test, and *one-way* ANOVA followed by LSD *post hoc* test. We calculated two-sided *p*-values to determine statistical significance. *P*<0.05 was considered statistically significant.
